# Sex differences in photon-counting computed tomography findings across hypertrophic phenotypes

**DOI:** 10.1093/ehjimp/qyag120

**Published:** 2026-06-29

**Authors:** Carmelo De Gori, Alberto Aimo, Yu Fu Ferrari Chen, Francesca Pignatelli, Mariaelena Occhipinti, Matilda Muca, Denisa Simona Zai, Alessandro Campora, Vincenzo Castiglione, Andrea Barison, Roberta Poletti, Silvia Maffei, Alberto Clemente, Giancarlo Todiere, Giuseppe Vergaro, Michele Emdin

**Affiliations:** Radiology Unit, Fondazione Toscana Gabriele Monasterio, Pisa and Massa, Italy; Interdisciplinary Center for Health Sciences, Scuola Superiore Sant’Anna, Cardiology Department, Fondazione Toscana Gabriele Monasterio, Piazza Martiri della Libertà 33, 56127 Pisa, Italy; Cardiology Department, Fondazione Toscana Gabriele Monasterio, via Moruzzi 1, 56124 Pisa, Italy; Interdisciplinary Center for Health Sciences, Scuola Superiore Sant’Anna, Cardiology Department, Fondazione Toscana Gabriele Monasterio, Piazza Martiri della Libertà 33, 56127 Pisa, Italy; Cardiology Department, Fondazione Toscana Gabriele Monasterio, via Moruzzi 1, 56124 Pisa, Italy; Radiology Unit, Fondazione Toscana Gabriele Monasterio, Pisa and Massa, Italy; Radiology Unit, Fondazione Toscana Gabriele Monasterio, Pisa and Massa, Italy; Radiology Unit, Fondazione Toscana Gabriele Monasterio, Pisa and Massa, Italy; Radiology Unit, Fondazione Toscana Gabriele Monasterio, Pisa and Massa, Italy; Interdisciplinary Center for Health Sciences, Scuola Superiore Sant’Anna, Cardiology Department, Fondazione Toscana Gabriele Monasterio, Piazza Martiri della Libertà 33, 56127 Pisa, Italy; Cardiology Department, Fondazione Toscana Gabriele Monasterio, via Moruzzi 1, 56124 Pisa, Italy; Interdisciplinary Center for Health Sciences, Scuola Superiore Sant’Anna, Cardiology Department, Fondazione Toscana Gabriele Monasterio, Piazza Martiri della Libertà 33, 56127 Pisa, Italy; Cardiology Department, Fondazione Toscana Gabriele Monasterio, via Moruzzi 1, 56124 Pisa, Italy; Interdisciplinary Center for Health Sciences, Scuola Superiore Sant’Anna, Cardiology Department, Fondazione Toscana Gabriele Monasterio, Piazza Martiri della Libertà 33, 56127 Pisa, Italy; Cardiology Department, Fondazione Toscana Gabriele Monasterio, via Moruzzi 1, 56124 Pisa, Italy; Cardiology Department, Fondazione Toscana Gabriele Monasterio, via Moruzzi 1, 56124 Pisa, Italy; Cardiovascular Endocrinology Unit, Fondazione Toscana Gabriele Monasterio, via Moruzzi 1, 56124 Pisa, Italy; Radiology Unit, Fondazione Toscana Gabriele Monasterio, Pisa and Massa, Italy; Interdisciplinary Center for Health Sciences, Scuola Superiore Sant’Anna, Cardiology Department, Fondazione Toscana Gabriele Monasterio, Piazza Martiri della Libertà 33, 56127 Pisa, Italy; Cardiology Department, Fondazione Toscana Gabriele Monasterio, via Moruzzi 1, 56124 Pisa, Italy; Interdisciplinary Center for Health Sciences, Scuola Superiore Sant’Anna, Cardiology Department, Fondazione Toscana Gabriele Monasterio, Piazza Martiri della Libertà 33, 56127 Pisa, Italy; Cardiology Department, Fondazione Toscana Gabriele Monasterio, via Moruzzi 1, 56124 Pisa, Italy; Interdisciplinary Center for Health Sciences, Scuola Superiore Sant’Anna, Cardiology Department, Fondazione Toscana Gabriele Monasterio, Piazza Martiri della Libertà 33, 56127 Pisa, Italy; Cardiology Department, Fondazione Toscana Gabriele Monasterio, via Moruzzi 1, 56124 Pisa, Italy

**Keywords:** photon-counting computed tomography, hypertrophic cardiomyopathy, transthyretin amyloid cardiomyopathy, left ventricular hypertrophy, sex differences, extracellular volume

## Abstract

**Aims:**

Photon-counting computed tomography (PCCT) enables simultaneous assessment of left ventricular morphology, function, and myocardial tissue characterization. Whether sex influences PCCT findings across hypertrophic phenotypes remains uncertain.

**Methods and results:**

We studied consecutive adults with a left ventricular hypertrophic phenotype undergoing PCCT. Patients were classified as hypertrophic cardiomyopathy (HCM), transthyretin amyloid cardiomyopathy (ATTR-CM), or secondary left ventricular hypertrophy (LVH). Sex differences in PCCT-derived left ventricular mass index, volumes, ejection fraction (EF), late iodine enhancement (LIE), and extracellular volume (ECV) were assessed overall and by aetiology. In patients with clinically indicated cardiovascular magnetic resonance (CMR) within ±12 months, sex effects on CT-CMR agreement were also evaluated. Among 182 patients, 50 were women and 132 were men; 83 had paired PCCT-CMR data. In the overall cohort, women had lower left ventricular mass index, smaller left ventricular volumes, higher EF, slightly lower ECV, and lower LIE extent than men. In HCM, women and men showed similar wall thickness, left ventricular mass, LIE burden, and ECV. In secondary LVH, women had smaller volumes and higher EF, without meaningful differences in tissue markers. In ATTR-CM, women appeared less remodelled and less infiltrative, although only four women were included. CT-CMR agreement for wall thickness, left ventricular mass, EF, scar extent, and ECV was similar in women and men. Diagnostic ECV thresholds were also consistent across sexes.

**Conclusion:**

Sex influences PCCT hypertrophic phenotypes mainly through remodelling and functional adaptation rather than major differences in tissue characterization. CT-CMR agreement and diagnostic ECV thresholds appear robust across sexes.

## Graphical Abstract

**Graphical Abstract:**
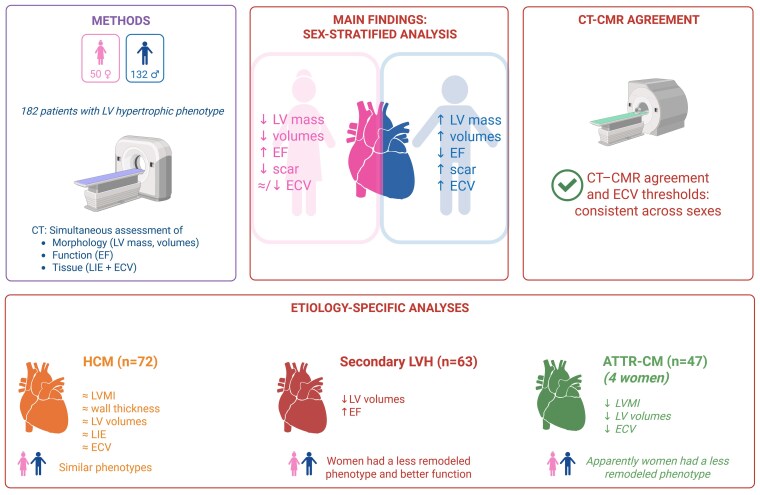

## Introduction

Photon-counting computed tomography (PCCT) combines high spatial resolution with spectral imaging and enables simultaneous characterization of ventricular morphology, function, and myocardial tissue composition, including late iodine enhancement (LIE) and extracellular volume (ECV).^1^ In a previous study by our group, PCCT showed strong agreement with cardiovascular magnetic resonance (CMR) for left ventricular (LV) mass, ejection fraction (EF), scar detection, and ECV, and differentiated hypertrophic cardiomyopathy (HCM), transthyretin amyloid cardiomyopathy (ATTR-CM), and secondary LV hypertrophy (LVH) on the basis of distinct morphologic and tissue profiles.^2^

A sex-stratified analysis is relevant because sex may influence cardiac remodelling, cavity size, systolic performance, and myocardial fibrosis, potentially affecting both disease expression and interpretation of quantitative imaging biomarkers.^3^ Establishing whether these differences are also identified by PCCT is important to better understand phenotype expression in women and men and to determine whether CT-CMR agreement and proposed ECV thresholds remain consistent across sexes. We therefore investigated whether PCCT-derived morphologic and tissue parameters, CT-CMR agreement, and diagnostic ECV thresholds differed between women and men.

## Methods

Consecutive adults with an LV hypertrophic phenotype who underwent PCCT at a tertiary referral centre between July 2024 and December 2025 were included. Patients were categorized as HCM, ATTR-CM, or secondary LVH according to guideline-based diagnostic criteria.^4^

All participants underwent cardiac PCCT using a standardized protocol including a delayed myocardial phase for tissue characterization. Quantitative PCCT variables included maximal wall thickness, LV volumes, EF, LV mass index (LVMI), LIE, iodine density, and ECV. Delayed-phase 40 keV virtual monoenergetic images and iodine maps were used for LIE and ECV analysis, and ECV was calculated with correction for same-day haematocrit. Image analysis was performed by blinded readers using the 17-segment model.

CMR scans performed as clinically indicated within ±12 months of PCCT were used for CT-CMR comparisons. CMR scans were re-read by blinded expert readers, and morphologic measurements were harmonized using the same contouring convention for LV mass. We compared women and men in the overall cohort and within each aetiologic subgroup, and evaluated whether sex influenced CT-CMR agreement or the performance of ECV thresholds.

LV mass and LV volumes were indexed to body surface area and are reported as LVMI (g/m^2^) and volume indices (mL/m^2^), respectively. Sex-specific normal ranges for LV mass were not used to define diagnosis or subgroup membership; LVMI was analysed as a continuous phenotype variable in sex-stratified and adjusted analyses.

Exploratory multivariable models adjusted for age and body MI (BMI) were used. Sex-specific ECV thresholds were explored *post hoc* by performing receiver operating characteristic (ROC) analyses separately in women and men. Optimal cut-offs were identified within each sex-specific subgroup using the Youden index. Bootstrap resampling was performed as an exploratory internal sensitivity analysis to assess the stability of the re-estimated thresholds and was not pre-specified; bootstrap ranges were reported only when resampling was feasible given subgroup size.

## Results

The analysis included 182 patients undergoing PCCT, of whom 50 were women and 132 were men. By diagnosis, the cohort comprised 72 patients with HCM (25 women, 47 men), 47 with ATTR-CM (4 women, 43 men), and 63 with secondary LVH (21 women, 42 men). A paired CT-CMR cohort was available in 83 patients, including 22 women and 61 men.

In the overall CT cohort, women had lower LVMI (68.5 vs. 84.0 g/m^2^, *P* < 0.001), lower LV end-diastolic volume (67 vs. 82 mL/m^2^, *P* < 0.001), lower LV end-systolic volume index (23.5 vs. 35 mL/m^2^, *P* < 0.001), and higher LVEF (64% vs. 55%, *P* < 0.001). Women also had slightly lower global CT-ECV (31% vs. 32%, *P* = 0.023) and lower scar burden as assessed by the number of LIE-positive segments (2 vs. 5, *P* < 0.001). In contrast, maximal wall thickness, LIE prevalence [36/50 women (72%) vs. 104/129 men (81%), *P* = 0.229], and iodine density did not differ clearly between sexes (*Table 1*).

**Table 1:** Comparison of PCCT findings between women and men in the whole cohort and by diagnosis

CT variable	Whole cohort			HCM			ATTR-CM			Secondary LVH		
	Women (*n* = 50)	Men (*n* = 132)	*P-*value	Women (*n* = 25)	Men (*n* = 47)	*P-*value	Women (*n* = 4)	Men (*n* = 43)	*P-*value	Women (*n* = 21)	Men (*n* = 42)	*P-*value
LVMI (g/m^2^)	69 (60–93)	84 (72–109)	**<0**.**001**	77 (64–101)	85 (75–111)	0.236	59 (55–71)	105 (83–122)	**0**.**014**	64 (59–75)	72 (60–83)	0.288
Maximal LV wall thickness (mm)	17 (14–20)	17 (15–20)	0.612	20 (17–22)	17 (17–21)	0.137	15 (14–16)	19 (18–22)	0.065	15 (13–16)	14 (13–16)	0.964
LVEDVi (mL/m^2^)	67 (60–79)	82 (70–102)	**<0**.**001**	70 (64–80)	78 (70–94)	0.131	64 (59–71)	89 (75–110)	**0**.**023**	62 (53–77)	79 (68–98)	**0**.**009**
LVESVi (mL/m^2^)	23.5 (18–30)	35 (26–51)	**<0**.**001**	25 (18–30)	29.5 (23–40)	0.077	26 (22–30)	41 (32–56)	**0**.**014**	20 (18–28)	35 (29–55)	**<0**.**001**
LVEF (%)	64 (61–70)	55 (48–65)	**<0**.**001**	64 (62–70)	62 (55–68)	0.076	62 (58–65.2)	52 (47–57)	0.118	66 (60–70)	54 (48–63)	**0**.**010**
LIE, number of patients (%)	36/50 (72)	104/129 (81)	0.229	24/25 (96)	44/47 (94)	1.000	3/4 (75)	42/43 (98)	0.165	9/21 (43)	18/39 (46)	1.000
LIE extent (*n* segments)	2 (1–4)	5 (2–15)	**<0**.**001**	2 (1–5)	4 (2–7)	0.133	11 (4–17)	17 (14–17)	0.259	1 (0–2)	1 (0–2)	0.317
ECV (%)	31 (27–34)	32 (28–42)	**0**.**023**	32.5 (27–34)	31 (28–36)	0.926	36.5 (36–39)	48 (41–54)	**0**.**032**	29 (25–31)	28 (26–30)	0.731
Iodine density (g/mL)	1.7 (1.3–1.8)	1.8 (1.5–2.2)	0.083	1.8 (1.7–2)	1.7 (1.5–1.9)	0.528	1.4 (1.1–1.8)	2.2 (1.9–2.5)	0.059	1.6 (1.3–1.7)	1.4 (1.4–1.7)	1.000

Data are reported as median (interquartile range). LV mass and LV volumes were indexed to body surface area. Sex-specific normal reference limits were not applied for classification purposes. LIE is reported as the number of patients with LIE/number with available data (%). *P*-values were calculated using the Mann–Whitney *U* test for continuous variables and Fisher’s exact test for categorical variables. Significant *P*-values are reported in bold. ATTR-CM, amyloid transthyretin cardiomyopathy; ECV, extracellular volume; LV, left ventricular; LVEDVi, left ventricular end-diastolic volume index; LVEF, left ventricular ejection fraction; LVESVi, left ventricular end-systolic volume index; LVH, left ventricular hypertrophy.

When analyses were stratified by diagnosis, HCM showed no significant sex-related differences in CT phenotype. Women and men with HCM had similar maximal wall thickness (20 vs. 17 mm, *P* = 0.137), LVMI (77 vs. 85 g/m^2^, *P* = 0.236), global CT-ECV (32.5% vs. 31.0%, *P* = 0.926), LIE extent (2 vs. 4 segments, *P* = 0.133), and LIE prevalence [24/25 women (96.0%) vs. 44/47 men (93.6%), *P* = 1.000]. Only borderline trends were observed for LV end-systolic volume index and LVEF (*Table 1*).

The clearest sex signal was observed in secondary LVH. Women had lower LV end-diastolic volume (62 vs. 80 mL/m^2^, *P* = 0.009), lower LV end-systolic volume index (20 vs. 36 mL/m^2^, *P* < 0.001), and higher LVEF (66% vs. 53.5%, *P* = 0.010) than men. These differences remained significant after exploratory multiple-testing correction (data not shown). In contrast, maximal wall thickness, LVMI, global CT-ECV, LIE burden, and LIE prevalence [9/21 women (43%) vs. 18/39 men (46%), *P* = 1.000] were not meaningfully different (*Table 1*).

In ATTR-CM, women appeared to have a less remodelled and less infiltrative phenotype, with lower LVMI, smaller LV volumes, and lower global CT-ECV than men (*Table 1*). LIE was present in 3/4 women (75%) and 42/43 men (98%). However, only four women with ATTR-CM were included, and this subgroup must therefore be considered descriptive only.

Adjusted analyses further supported heterogeneity of sex effects across aetiologies. After accounting for age and BMI, there was a significant sex-by-diagnosis interaction for global CT-ECV (*P* = 0.016), with borderline interactions for maximal wall thickness (*P* = 0.050) and LVMI (*P* = 0.074).

No significant differences emerged between men and women in the prevalence of crypts, left anterior descending myocardial bridging, apical aneurysm, or overall coronary artery disease burden, either in the whole cohort or within the aetiologic subgroups; the only significant finding in the overall cohort was a higher frequency of apicalized papillary muscle insertion in women (data not shown).

In the paired cohort, median CT-CMR differences for wall thickness, LVMI, LVEF, and scar extent were essentially zero in both sexes. Agreement for the presence of LIE/LGE was observed in 22/22 women and 58/59 men, corresponding to 100.0% and 98.3%, respectively. These estimates should be interpreted alongside the absolute subgroup sizes, particularly in women. Among the 31 patients with paired CT-CMR ECV data, there was no significant sex difference in CT-CMR ECV bias, and the same overall message was confirmed when the analysis was restricted to studies performed within 90 days (11 women and 36 men for paired PCCT-CMR data; 6 women and 13 men for paired ECV data; *Table 2*).

**Table 2:** Agreement between PCCT and CMR

Variable	Women	Men	*P-*value
Paired PCCT-CMR cohort (within ±12 months)			
Patients with paired PCCT and CMR, *n*	22	61	
Maximal wall thickness bias, PCCT-CMR (mm)	0 (0–0)	0 (0–0)	0.341
Maximal wall thickness absolute error (mm)	0 (0–0)	0 (0–1)	0.770
LVMI bias, PCCT-CMR (g/m^2^)	0.0 (−1.0 to 8.2)	0.0 (−1.0 to 5.8)	0.772
LVMI absolute error (g/m^2^)	2.0 (0.2–8.2)	3.0 (0.0–8.0)	0.801
LVEF bias, PCCT-CMR (%)	0.0 (0.0–3.5)	0.0 (−0.8 to 2.0)	0.597
LVEF absolute error (%)	1.0 (0.0–4.0)	1.0 (0.0–4.0)	0.859
Scar extent bias (segments with LIE/LGE)	0 (0–0)	0 (0–0)	0.784
Scar extent absolute error (segments)	0 (0–0)	0 (0–0)	0.17
Any LIE/LGE agreement, % (*n*/*N*)	100.0 (22/22)	98.3 (58/59)	0.997
Paired global ECV datasets, *n*	10	21	
Global ECV bias, PCCT-CMR (%)	−2.0 (−3.8 to 3.0)	−1.0 (−7.0 to 0.0)	0.691
Global ECV absolute error (%)	3.0 (3.0–6.2)	4.0 (1.0–8.0)	0.995
Sensitivity analysis (PCCT-CMR interval ≤90 days)			
Patients with paired PCCT and CMR, *n*	11	36	
LVMI absolute error (g/m^2^)	1.0 (0.0–6.0)	3.0 (0.0–8.0)	0.604
LVEF absolute error (%)	1.0 (0.0–3.0)	1.0 (0.0–3.0)	0.862
Any LIE/LGE agreement, % (*n*/*N*)	100.0 (11/11)	97.1 (34/35)	1.000
Paired global ECV datasets, *n*	6	13	
Global ECV bias, PCCT-CMR (%)	−2.0 (−3.8 to 2.0)	−6.0 (−11.0 to −1.0)	0.363
Global ECV absolute error (%)	3.0 (3.0–3.8)	7.0 (5.0–11.0)	0.202

Women are compared with men in the overall paired cohort and in the sensitivity subset with PCCT-CMR interval ≤90 days. Continuous variables are median (interquartile range). The bias was calculated as the difference between the same measure obtained through PCCT or CMR. For the overall paired cohort, the *P* values for LVMI absolute error, LVEF absolute error, and global ECV bias follow the original sex-analysis summary; the remaining continuous-variable *P* values were derived from the paired dataset using Mann–Whitney *U* tests, and agreement percentages were compared using Fisher’s exact tests. ECV, extracellular volume; LGE, late gadolinium enhancement; LIE, late iodine enhancement; LVEF, left ventricular ejection fraction; LVMI, left ventricular mass index.

For differentiating ATTR-CM from secondary LVH, the previously derived ECV threshold of 34%^2^ performed well in both sexes, and sex-specific re-estimation yielded nearly identical cut-offs (34% in women, 35% in men). In women, this comparison was based on 4 ATTR-CM and 21 secondary LVH patients; in men, it was based on 43 ATTR-CM and 42 secondary LVH patients. For HCM vs. secondary LVH, discrimination remained only modest in both sexes, with no meaningful threshold shift. This comparison included 25 women and 47 men with HCM, and 21 women and 42 men with secondary LVH. For ATTR-CM vs. HCM, apparent sex-related threshold differences were driven largely by the very small number of women with ATTR-CM, and formal ECV-by-sex interaction tests were not significant (*Table 3*).

**Table 3:** Sex-specific diagnostic performance of global PCCT ECV thresholds

Comparison	Published threshold (%)	Women AUC	Women sens. (%)	Women spec. (%)	Men AUC	Men sens. (%)	Men spec. (%)	Women re-est. threshold (%)	Men re-est. threshold (%)	Women bootstrap (%)	Men bootstrap (%)	ECV × sex interaction *P*-value
ATTR-CM vs. secondary LVH	≥34	0.96	100	90	0.98	90	98	34	35	34–37	32–41	0.55
HCM vs. secondary LVH	≥32	0.67	54	76	0.70	46	90	33	32			0.53
ATTR-CM vs. HCM	≥40	0.84	25	100	0.92	83	91	34	38	34–43		0.65

Previously derived thresholds are from the parent study; re-estimated thresholds are exploratory sex-specific optimal cut-offs derived from *post hoc* ROC analyses using the Youden index. Bootstrap ranges represent exploratory internal resampling analyses and were not pre-specified; they are reported only when feasible. ATTR-CM comparisons in women should be interpreted cautiously because only four women were included. HCM, hypertrophic cardiomyopathy; LVH, left ventricular hypertrophy.

## Discussion

This sex-stratified analysis provides three main findings. First, women in the overall cohort exhibited a smaller-remodelling and higher-function PCCT profile, characterized by lower LV mass, smaller LV volumes, and higher EF. Secondly, this pattern was not uniform across aetiologies: it was most evident in secondary LVH, largely absent in HCM, and only descriptively present in ATTR-CM because of the very limited number of affected women. Thirdly, sex did not materially influence CT-CMR agreement or the diagnostic performance of ECV thresholds.

The finding of a less remodelled phenotype in women is biologically plausible and supports the interpretation that sex-related variation in hypertrophic phenotypes is driven predominantly by differences in ventricular remodelling and systolic adaptation, rather than by substantial differences in myocardial tissue composition. Although women had slightly lower global ECV and lower scar extent overall, the most robust differences involved LV mass, end-diastolic and end-systolic volumes, and EF.

The absence of major sex differences in HCM is clinically relevant. Women and men with HCM showed comparable wall thickness, LIE burden, and ECV values, suggesting that once overt HCM is established, the myocardial phenotype identified by PCCT is broadly similar across sexes. This supports a common interpretative framework for PCCT-derived fibrosis and ECV metrics in women and men with HCM. In contrast, in secondary LVH, sex-related variation was dominated by differences in ventricular geometry and functional adaptation rather than tissue characteristics. Women had smaller ventricular volumes and higher EF, without meaningful differences in wall thickness, LIE, or ECV. This suggests that in pressure- or load-related hypertrophy, sex may be more relevant for interpreting remodelling and functional indices than fibrosis-related markers. The ATTR-CM subgroup deserves cautious interpretation. Women appeared to have a less remodelled and less infiltrative phenotype, but the very small number of female patients precludes firm conclusions. These findings should therefore be viewed as hypothesis-generating and warrant confirmation in larger, preferably multicentre cohorts with better female representation.

Another important observation is the consistency of CT-CMR agreement across sex. This strengthens the generalizability of the main study findings and supports the use of PCCT as a robust alternative when CMR is contraindicated. Similarly, the lack of a meaningful sex effect on diagnostic ECV thresholds indicates that disease-related myocardial biology is a stronger determinant of ECV discrimination than sex itself and does not support sex-specific recalibration of the proposed thresholds.

Several limitations must be acknowledged. This was an exploratory *post hoc* subgroup analysis and was not specifically powered to detect sex differences within each aetiologic category. The marked sex imbalance, particularly in ATTR-CM, limits the robustness of subgroup comparisons and increases the risk of over-interpreting descriptive trends. This imbalance may partly reflect referral bias and possible under-diagnosis of women with ATTR-CM or HCM and could therefore influence the apparent sex-stratified phenotypes observed in this cohort. In addition, some observed differences may partly reflect body size, disease stage, or referral patterns that cannot be fully addressed in this analysis.

## Conclusion

In conclusion, sex appears to influence hypertrophic phenotypes on PCCT mainly through remodelling and functional adaptation rather than major differences in fibrosis-related tissue characterization. The most consistent sex-related differences were observed in secondary LVH, whereas HCM showed substantial similarity between women and men, and ATTR-CM requires further study in larger cohorts. Sex did not affect CT-CMR agreement or justify sex-specific ECV thresholds. These findings support PCCT as a robust imaging tool across sexes, while suggesting that sex-based interpretation may be most relevant for ventricular geometry and function rather than tissue characterization.

## Data Availability

The data underlying this article will be shared on reasonable request to the corresponding author.

## Lead author biography

Dr Alberto Aimo, MD, PhD, FESC, FHFA, is a cardiologist at the Fondazione Toscana Gabriele Monasterio, a division of the Italian National Research Council, and a researcher at the Scuola Superiore Sant’Anna in Pisa, Italy. His research primarily focuses on heart failure and cardiomyopathies, with an emphasis on the use of biomarkers and advanced imaging for diagnosis and risk stratification, as well as drug and device therapies and structural interventions in heart failure.
